# 3D PRINTING IN COMPLEX TIBIAL FRACTURE CLASSIFICATION & PLANNING

**DOI:** 10.1590/1413-785220243203e269705

**Published:** 2024-08-02

**Authors:** Fuyang Chen, Chenyu Huang, Chen Ling, Jinming Zhou, Yufeng Wang, Po Zhang, Xiao Jiang, Xiaoming Xu, Jian Jian, Jiayi Li, Liming Wang, Qingqiang Yao

**Affiliations:** 1 Nanjing Medical University, Department of Orthopaedic Surgery, Nanjing First Hospital, Nanjing, China.; 2 Nanjing Medical University, Institute of digital medicine, Nanjing, China.; 3 Nanjing Medical University, Institute of Digital Medicine, Key Lab of Additive Manufacturing Technology, Nanjing, China.; 4 Department of Orthopaedic Surgery, Pukou Hospital, Pukou branch of Jiangsu Province Hospital, Nanjing, China.; 5 Univerisity of California, Department of Biomedical Engineering, Irvine, USA.

**Keywords:** 3D Printing, Tibial Plateau Fracture, Anatomic Landmarks, Preoperative Care, Impressão 3D, Fratura do Planalto Tibial, Marcos anatômicos, Cuidados pré-operatórios

## Abstract

**Objective:**

Tibial plateau fractures are common intra-articular fractures that pose classification and treatment challenges for orthopedic surgeons.

**Objective:**

This study examines the value of 3D printing for classifying and planning surgery for complex tibial plateau fractures.

**Methods:**

We reviewed 54 complex tibial plateau fractures treated at our hospital from January 2017 to January 2019. Patients underwent preoperative spiral CT scans, with DICOM data processed using Mimics software. 3D printing technology created accurate 1:1 scale models of the fractures. These models helped subdivide the fractures into seven types based on the tibial plateau's geometric planes. Surgical approaches and simulated operations, including fracture reduction and plate placement, were planned using these models.

**Results:**

The 3D models accurately depicted the direction and extent of fracture displacement and plateau collapse. They facilitated the preoperative planning, allowing for precise reconstruction strategies and matching intraoperative details with the pre-printed models. Post-surgery, the anatomical structure of the tibial plateau was significantly improved in all 54 cases.

**Conclusion:**

3D printing effectively aids in the classification and preoperative planning of complex tibial plateau fractures, enhancing surgical outcomes and anatomical restoration. *Level of Evidence IV, Prospective Study.*

## RESUMO

**Objetivo:**

As fraturas do planalto tibial são fraturas intra-articulares comuns de classificação e tratamento desafiadores aos cirurgiões ortopédicos.

**Objetivo:**

Este estudo investiga o uso de impressão 3D para classificar e planejar a cirurgia de fraturas complexas do planalto tibial.

**Métodos:**

54 fraturas complexas do planalto tibial tratadas em nosso hospital de janeiro de 2017 a janeiro de 2019 foram revisadas. Os pacientes foram submetidos a tomografias computadorizadas em espiral pré-operatórias, com dados DICOM processados usando o software Mimics. A tecnologia de impressão 3D gerou modelos precisos em escala 1:1 das fraturas. Estes modelos ajudaram a subdividir as fraturas em sete tipos com base nos planos geométricos do planalto tibial. As abordagens cirúrgicas e as operações simuladas, incluindo a redução da fratura e a colocação de placa, foram planejadas utilizando estes modelos.

**Resultados:**

Os modelos 3D representaram com precisão a direção e a extensão da deslocação da fratura e do colapso do planalto. Os modelos facilitaram o planejamento pré-operatório, viabilizando estratégias de reconstrução precisas e a correspondência dos detalhes intraoperatórios com os modelos pré-impressos. Após a cirurgia, a estrutura anatômica do planalto tibial melhorou significativamente em todos os 54 casos.

**Conclusão:**

A impressão 3D ajuda na classificação e no planejamento pré-operatório de fraturas complexas do planalto tibial, melhorando os resultados cirúrgicos e a restauração anatômica. Nível de Evidência IV, Estudo Prospectivo.

## INTRODUCTION

 Tibial plateau fracture is one of the common intra-articular fractures in the clinic. And its accurate classification and treatment is a complex problem for orthopedic surgeons. ^1^ At present, the commonly used clinical classification method of tibial plateau fracture was put forward by Schatzker et al. ^2^ in 1949, which is based on X-ray. It does not consider the displacement of fracture in the sagittal position. So sometimes, the classification can not effectively guide the formulation of the treatment plan, especially in the case of the posterior tibial plateau. In 2009, Professor Luo Congfeng ^3^ proposed a three-column classification method of tibial plateau fractures based on CT, which divides the tibial plateau into lateral column region, medial column region, and posterior column region. However, this classification method can not directly reflect the degree of comminution and collapse of the articular surface of the fracture. Also, this classification method can not guide the design of preoperative operation well. Complex tibial plateau fractures are often associated with two-column or three-column fractures. Good preoperative planning can significantly shorten the operation time and improve the restoration effect. With the emergence of 3D printing digital medical technology, accurate and individualized treatment has become the trend of orthopedic trauma surgery. ^4^ Different literature reviews have revealed an increasing use of 3D-printed models in surgery, ^5^ orthopedics ^6^ and orthopedic trauma, ^7^ interventional radiology, ^8^ surgical teaching and assessment, ^9^ etc. The reported advantages of 3DP-based approaches refer to the robust capabilities of customization based on patient imagistic data (computer tomography (CT), magnetic resonance imaging (MRI)), improved visualization of the anatomy allowing better diagnosis evaluation, a decrease in operating time, and radiologic exposure during surgery, improved intervention accuracy, and enhanced communication among physicians and with patients. ^5^^,^^7^

## METHODS

### Inclusion and exclusion criteria for cases

Inclusion criteria: ①cases of unilateral closed tibial plateau fracture treated by surgery in the department of orthopedics of our hospital during 2017.01-2019.01. ② complex tibial plateau fractures with double-column or three-column injuries. ③The time from injury to operation was less than 14 days.

Exclusion criteria: ①patients with chronic lesions of knee joint and knee joint dysfunction before the injury. ②complicated with vascular and nerve damage on the affected side. ③those with serious underlying diseases or unable to cooperate with treatment.

### General information

A total of 54 patients were included in this study, including 36 males and 18 females, with an average age of 50.2 ± 2.8 years old. According to the theoretical basis of the three-column classification of the tibial plateau, combined with 3D printing model classification, there were 18 cases of type Ⅳ fracture, 13 cases of type Ⅴ fracture, 8 cases of type Ⅵ fracture, and 15 cases of type Ⅶ fracture. All cases were treated with internal fixation. The time from injury to operation was 12 days, with an average of 6.4 ± 1.5 days. The Ethics Committee has approved this study of our hospital. All patients have signed the informed consent form for the operation.

### Ethics approval and consent to participate

All procedures performed in studies involving human participants were by the ethical standards of the institutional and/or national research committee and with the 1964 Helsinki Declaration and its later amendments or comparable ethical standards. Informed consent was obtained from all individual participants included in the study.

### Using 3D printing model to refine the classification of plateau fractures from the geometric plane 3-column 3-zone typing method

3D model printing: all the 54 cases underwent 64-slice spiral CT thin-slice scanning (0.6mm) before the operation, and the DICOM data were input into the computer. The Mimics software was used to process the data. And the 3D printing technique was used to print the three-dimensional model of the fracture (1:1).

**Figure 1.**
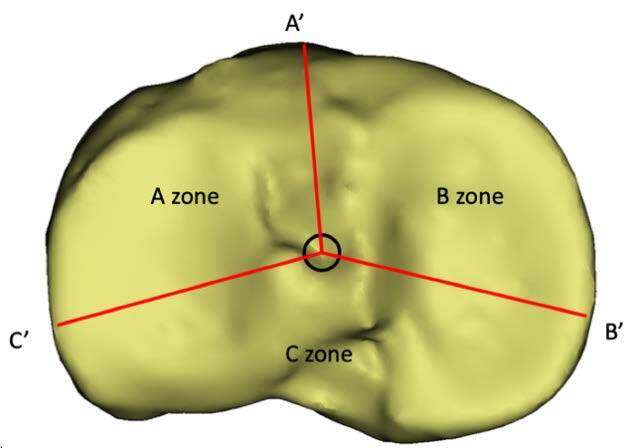
Classification of the 3 zones.

 Based on the classification of the geometric plane of the platform ( Figure 1 ), the overlooking view of the tibial plateau shows that the O point is the midpoint of the tibial spine line. The A’ point is the tibial tubercle. The B’ point is the medial crest of the tibial plateau. And the C ‘point is the anterior edge of the fibular head. The tibial plateau is divided into three plane parts by OA’, OB’ and OC,’ which are defined as A zone, B zone, and C zone, respectively. ( Table 1 ) 

**Table 1.** Three-column and three-zone classification of tibial plateau fractures.

Classification	Zone	Position	Approach
I	A	Supine position	Anterolateral approach
II	B	Supine position	Anterior medial approach
III	C	Prone position	Posterior approach
IV	A+B	Supine position	Combined medical and lateral approach
V	A+C	Supine position	Anterolateral peroneal approach
VI	B+C	Supine position	Posteromedial approach
VII	A+B+C	Floating position	Anterolateral approach + posteromedial approach

Type IV-VII are included in this project.

O: Midpoint of tibial spine line A’:tibial tuberosity; B’:Medial spine of tibial plateau; C’: Anterior margin of the fibular head.

**Figure 2.**
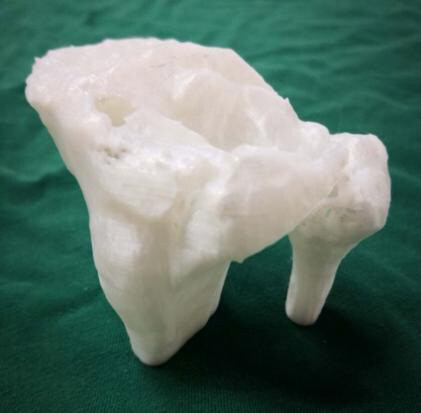
3D printed tibial plateau fracture model.

### Preoperative planning and surgical simulation steps

 ① The CT data of patients with quasi-fracture were processed on a particular computer platform. And then, the tibial plateau fracture model was printed 1:1 ( Figure 2 ). The printing machine is the selective laser sintering equipment Farsoon401. The printing material is nylon powder material for laser sintering. 

② The fractures were re-classified by three-column classification according to the fracture imaging data and 3D visual model to achieve accurate classification according to the principle.

③ Evaluate the displacement direction of the bone fracture block. And through the direction, distribution, and movement of the fracture block, we can accurately evaluate the displacement direction of the bone fracture block.

④ Evaluate the collapse site of the articular surface. And through the detailed analysis and observation of the articular surface, we can determine the actual collapse site of the articular surface.

⑤ Establish the surgical approach. By analyzing the fractured mass and articular surface of the complex tibial plateau fracture, the intraoperative approach can be established to create conditions for reducing trauma injury.

⑥ Determine the position and the number of steel plate implantation. Predicting the position and number of steel plate implantation and the pre-bending data of steel plate before operation can effectively reduce the operation time, trauma injury, and the use of anesthetic drugs.

⑦ Simulate the operation. The reduction of the fracture block and the placement of the steel plate can be performed on the 3D model according to the operation plan to improve the operation’s proficiency.

### Preoperative scheme design of complex tibial plateau fracture (type IV-VII)

**Figure 3.**
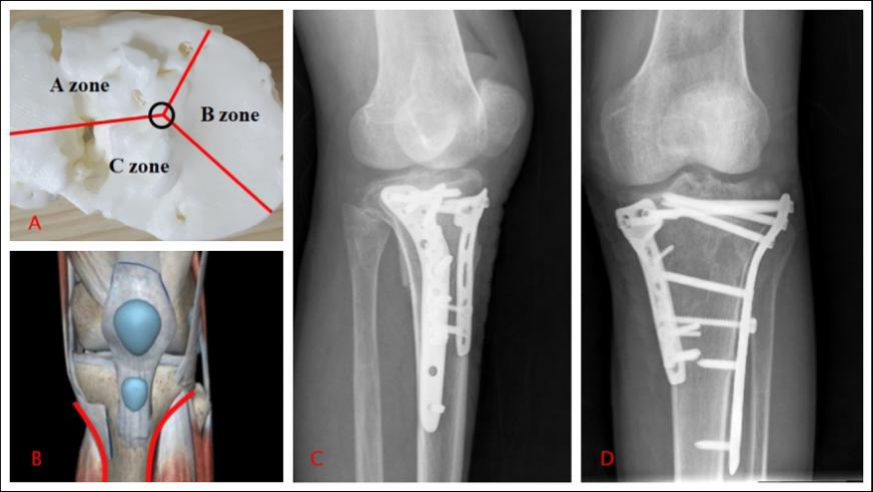
Type IV tibial plateau fracture.

 Type IV: supine position, choice of surgical approach: combined medial and lateral approach. ( Figure 3 ). 

**Figure 4.**
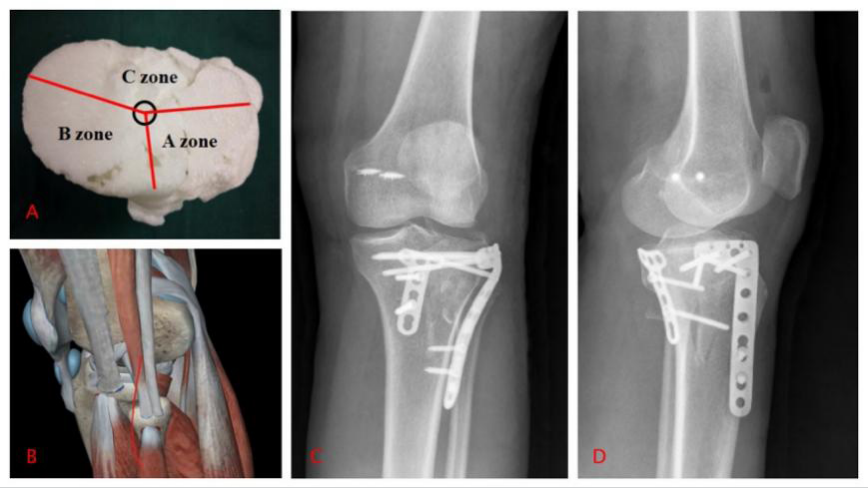
Type V tibial plateau fracture.

 Type Ⅴ: supine position, choice of surgical approach: an anterolateral peroneal head approach to fix the lateral column, and whether to fix it according to the stability of the posterior column ( Figure 4 ). 

**Figure 5.**
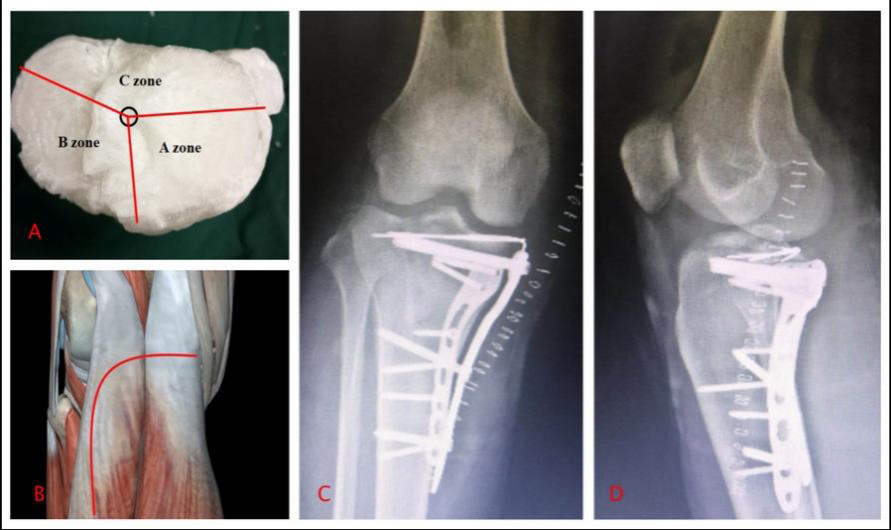
Type VI tibial plateau fracture.

 Type VI: supine position, choice of surgical approach: modified posterior medial approach ( Figure 5 ). 

**Figure 6.**
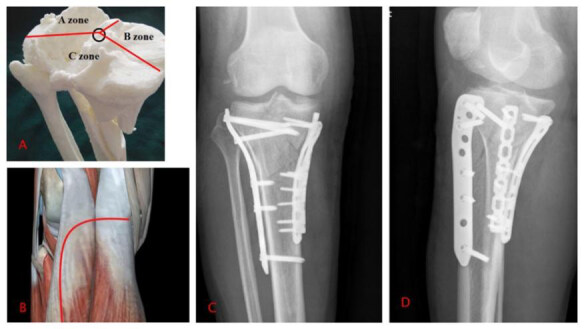
Type VII tibial plateau fracture.

 Type Ⅶ: floating position, choice of surgical approach: the meniscus was repaired by the anterolateral approach, the anterolateral approach fixed the lateral column, and the posteromedial approach fixed the medial column and the posterior column. ( Figure 6 ) 

A: 3D printed model; B: Approach; C, D: X-rays after the operation.

A: 3D printed model; B: Approach; C, D: X-rays after the operation.

A: 3D printed model; B: Approach; C, D: X-rays after the operation.

A: 3D printed model; B: Approach; C, D: X-rays after the operation.

### Surgical methods and postoperative management

 All patients were treated with general anesthesia or combined block anesthesia. After successful anesthesia, the affected limb was bound with a tourniquet with a pressure of 50KPA for 90 minutes. Antibiotics were routinely used before operation. Combined with preoperative classification, the operation was performed according to the surgical approach, fracture reduction mode, plate preshaping, screw direction, and length designed before operation. During the operation, the mode and amount of bone graft were determined according to the collapse of the articular surface. Explore the articular surface to repair meniscus and ligament injuries that need one-stage surgical repair as far as possible. ^10^ Intraoperative C-arm fluoroscopy confirmed the degree of fracture reduction and articular surface elevation. After the operation, the negative pressure drainage tube was routinely indwelled and removed within 48 hours. Prophylactic use of antibiotics was used within 48 hours after the operation. The contraction exercise of the quadriceps femoris was performed on the 3rd day after the operation. The flexion and extension function of the knee joint began to exercise 1 week later, and partial weight-bearing began to be carried out six weeks after the operation. And after the fracture healing was confirmed by X-ray, three months after the operation, patients began to bear weight completely gradually. 

### Observation indicators

#### The coincidence rate between the preoperative planning and the final surgical plan

Each case of complex platform fracture was designed according to the 3D printing model before operation. The specific surgical approach and implant scheme were designed. The specific operation plan was recorded. The consistency rate between the final operation plan and the preoperative plan was obtained.

#### Knee joint function score

 The patients were followed up for one year. During the follow-up, the anterior and lateral films of the knee joint were taken, and the knee joint function was evaluated by HSS score. ^11^ HSS score > 85 was excellent, 70-84 was good, 60-69 was fair, and < 59 was poor. 

#### Fracture healing time and postoperative complications

The fracture healing time of each case was recorded in the follow-up. And whether there were postoperative complications such as infection, screw fracture, and other complications were recorded.

#### Statistical processing

The above data were analyzed by SPSS18.0 statistical software. χ 2 test was used for the Comparison of counting data. T-test was used for the Comparison of measurement data. The difference was statistically significant (P < 0.05).

## RESULTS

### The coincidence rate between the preoperative planning and the final surgical plan

The classification of tibial plateau fracture combined with 3D printing is basically consistent with what was seen during the operation. The fracture type and articular surface collapse seen during the operation are basically the same as the model, which is of great help to the understanding and operation of the operation. In all 54 cases, the final operation plan of 51 cases was consistent with the design before operation. And 3 cases of them are type VII fracture. Before the operation, the lateral column was fixed by anterolateral approach, and the medial column and posterior column were fixed by the posteromedial approach. The bone mass of the lateral column was reduced and fixed by an anterolateral approach. It was found that the stability of the posterior column was good. The coincidence rate between the preoperative planning and the final surgical plan was 94.4% (the final plan was consistent with the preoperative planning and design of the number of cases / total number of cases * 100%). The postoperative CT results showed that the collapse of the articular surface of the platform was well reduced in all 54 patients.

### Knee joint function score

 During the follow-up six months after the operation, the excellent and good rate of knee joint function was 87.0%. And the excellent and good rate of knee joint function 12 months after the operation was 90.7%. Only one patient had poor knee joint function six months after the operation. And his knee joint function was improved after functional rehabilitation exercise. ( Table 2 ) 

**Table 2.** Comparison of knee joint function.

		Six month after the surgery	12 months after surgery
Case numbers		54	54
HSS score		88.1±6.1	88.4±6.9
knee joint function	Excellent	38(70%)	44(80%)
	Good	9(17%)	5(10%)
	Average	6(11%)	5(10%)
	Bad	1(2%)	0

(P > 0.05).

### Fracture healing time and postoperative complications

Postoperative follow-up showed that all 54 patients healed well. And the average healing time was 3.4±0.5 months. All 54 patients had no infection and no internal fixation loosening.

## DISCUSSION

### The importance of correct classification of tibial plateau fractures and preoperative planning

 The treatment of complex tibial plateau fractures has always been a difficult problem in orthopedics. ^12^ It is often accompanied by ligament, meniscus injury, and serious damage of nerve, blood vessel, and soft tissue, making fracture treatment more difficult. ^13^ Patients often have varying degrees of dysfunction after the operation, which seriously affects their ability to work. How to deal with complex tibial plateau fractures, reduce fracture complications, and make a good recovery of knee joint function. 

 The correct classification and preoperative planning of tibial plateau fractures are very important for selecting surgical approaches and fixation methods. CT scanning can scan the tibial plateau in axial, coronal, and sagittal planes to show the full picture of the tibial plateau. ^14^^-^^16^ At the same time, 3D printing can have a more intuitive understanding of the whole tibial plateau and the details of the fracture, which makes various measurements more convenient. Thus, we can have a quantitative understanding of the degree of fracture collapse and splitting. Also, it can provide a better reference for surgical approach and internal fixation placement. ^17^

### Development of classification methods for tibial plateau fractures

 A good classification method of tibial plateau fracture not only needs to accurately reflect the degree of fracture injury but also needs to guide clinical treatment. Hohl-Moore classification is a classification method based on X-ray, which divides tibial plateau fractures into five kinds of primary fractures and five kinds of fracture and dislocation. However, this method can not accurately reflect the degree of soft tissue injury during fracture, and it is difficult to guide clinical treatment. Schatzker classification increases the type of VI fracture with separation of metaphysis and diaphysis, widely used in the clinic. However, it also has disadvantages. In practical clinical Applications, simple collapse fracture is rare and can not be effectively distinguished. The fracture of the posterior lateral column of the tibial plateau is not distinguished, which is easy to cause missed diagnosis and affect the choice of surgical treatment. The content of the AO classification is detailed, which is conducive to academic research and communication. However, the content is too complex to remember. Also, it can not accurately reflect the relationship between articular surface collapse and fracture severity. Thus, it is not competent to guide the choice of clinical treatment and prognosis. ^18^ Based on the above, Luo Congfeng et al. suggested the classical three-column classification method based on three-dimensional CT. They analyzed the shape and location of the fracture from a three-dimensional point of view for the first time. ^3^ According to the division, it is divided into internal, external, and posterior three columns, which can accurately guide the surgical approach. But this classification did not put forward the concept of posteromedial and posterolateral tibial plateau fractures. Mao Yujiang et al. put forward the theory of “four columns and four quadrants” based on three columns. This classification distinguishes the morphological differences between posteromedial and posterolateral column fractures and is of great significance for the guidance of clinical posterior column fractures. ^19^

 With the emergence of 3D printing technology, we can better obtain the solid model of the fracture site before operation. Compared with the solid model, the surgeon has a deeper understanding of the local structure of the fracture. Based on the accurate classification, it is of greater significance to guide clinical surgery. ^20^^-^^22^

### Advantages of 3D printing technique in classification and preoperative planning of complex tibial plateau fractures

 Compared with CT scanning, 3D printing provides more details about proximal tibial bone and fracture, even the internal details of fracture. ^6^^-^^7^ 3D printing can intuitively classify and fix the fracture as a more detailed preoperative plan, guide the mode of operation, surgical approach, and fixation, providing better preoperative guidance for clinical practice. ^23^ And it can provide the possibility to better improve the classification of tibial plateau fracture. It has the following three advantages: 

Psychological advantage: After printing the 3D model of complex tibial plateau fracture, the operator can intuitively understand the fracture state and displacement. So that the surgeon can have more time and more intuitive to design the operation plan, to get twice the result with half the effort.

 Operational advantages: According to the operation on the simulated 3D model before the operation, the plates and screws can be implanted smoothly during the operation. For medial and lateral plateau fractures, we can often see the articular surface of the plateau; the reduction of the articular surface is not very difficult. Still, it may not be easy to see the posterior articular surface during operation in complex plateau fractures involving posterior column fractures. Therefore, the preoperative surgical design, the preshaping of the steel plate, and the direction and length of the screw are of great help to the operation. It greatly simplifies the difficulty of the operation, shortens the operation time, and reduces surgical trauma injury. ^24^^-^^25^

Communication advantages: before the operation, we use 3D printed models to communicate with patients. So that patients have a better understanding of the severity of their fractures and a certain understanding of the surgical plan and the expected effect of treatment. In this way, it can also eliminate the patients’ fear. And the compliance will be better.

With the combination of digital medicine and orthopedics, 3D printing technology can transform virtual 3D images into realistic three-dimensional objects, facilitating precise treatment for surgeons. 3D printed models applied to tibial plateau fractures have the characteristics of optimizing preoperative planning, providing individualized and precise treatment, which can reduce the difficulty of surgery and improve the accuracy of surgery.

Before surgery, the surgeons can accurately gauge the plate and screw, design the relevant angle and length with the aid of a computer. Simulated surgical operations can increase the operator’s operational proficiency. At the same time, it can intervene in advance to deal with the operation’s key areas and difficult points, which can reduce the risk of damaging blood vessels and nerves during the nail placement. The anatomical structure of the patient’s tibia and the adjacent relationship can be clarified by comparing and observing the physical model in real-time during surgery, reducing errors in judgment and surgery-related complications due to anatomical deformities and individual differences. The number of secondary surgeries is reduced, and the difficulty and risk of surgery are reduced. Only one verification fluoroscopy is required after nail placement, which greatly reduces operative time, x-ray fluoroscopy, and surgical bleeding.

In summary, 3D printing technology can further refine the classification of complex tibial plateau fractures. The surgical approach and internal fixation plan are planned through the solid model, which is intuitive and accurate. And the preoperative planning scheme is feasible, which greatly simplifies the operation and reduces surgical trauma injury. Also, it can be used as a routine item of preoperative preparation. In the future, under the guidance of a 3D printing template, the optimized preoperative planning of tibial plateau fracture and accurate internal fixation operation will be better applied to the clinic.

## Data Availability

The datasets used and/or analyzed during the current study are available from the corresponding author on reasonable request.
